# NASA Global Daily Downscaled Projections, CMIP6

**DOI:** 10.1038/s41597-022-01393-4

**Published:** 2022-06-02

**Authors:** Bridget Thrasher, Weile Wang, Andrew Michaelis, Forrest Melton, Tsengdar Lee, Ramakrishna Nemani

**Affiliations:** 1 Climate Analytics Group, Boulder, CO 80305 USA; 2 NASA Ames Research Center, Moffett Field, CA 94035 USA; 3 NASA ARC-CREST, CSUMB, Seaside, CA USA; 4 NASA Headquarters, Washington, DC USA

**Keywords:** Climate and Earth system modelling, Research data, Atmospheric science

## Abstract

We describe the latest version of the NASA Earth Exchange Global Daily Downscaled Projections (NEX-GDDP-CMIP6). The archive contains downscaled historical and future projections for 1950–2100 based on output from Phase 6 of the Climate Model Intercomparison Project (CMIP6). The downscaled products were produced using a daily variant of the monthly bias correction/spatial disaggregation (BCSD) method and are at 1/4-degree horizontal resolution. Currently, eight variables from five CMIP6 experiments (historical, SSP126, SSP245, SSP370, and SSP585) are provided as procurable from thirty-five global climate models.

## Abstract (summary)

**Table:** 

Measurement(s)	temperature of air • volume of hydrological precipitation • humidity • stellar radiation • atmospheric wind speed
Technology Type(s)	statistical downscaling
Sample Characteristic - Environment	atmosphere

## Background & Summary

The first version of the NASA Earth Exchange Global Daily Downscaled Projections^1^ archive (NEX-GDDP) was originally released in 2015 with the intention of enhancing public understanding and assisting the scientific community in the study of climate change impacts over scales from regional to local. Since then, the projections have been used by a variety of groups across the globe, from government researchers to students, from non-governmental organizations to industry professionals. While this original archive was based on the global climate model (GCM) output from the Climate Model Intercomparison Project Phase 5 (CMIP5), results from the state-of-the-art CMIP6 experiments have recently become available. The release of these newer climate projections has resulted in a strong interest from the community in a new version of NEX-GDDP.

**Fig. 1:**
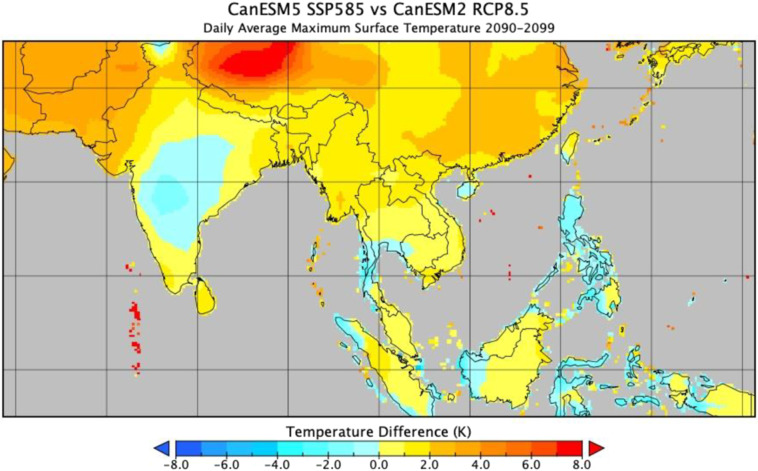
End of century daily average maximum near-surface air temperature difference between the downscaled CanESM5 model output (CMIP6) and the corresponding downscaled CanESM2 model output (CMIP5).

**Fig. 2:**
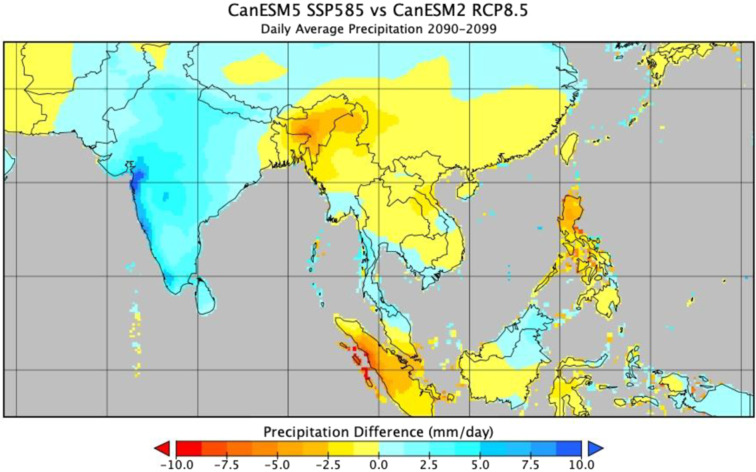
Same as Fig. 1, but for precipitation.

Here we present the latest version of the NEX-GDDP archive (NEX-GDDP-CMIP6). Not only are these updated projections based on the newer CMIP6 output, but they also include an expanded set of variables as shown in Table 1. Figures 1 and 2 show examples of the differences between corresponding model outputs from the two archives. The downscaling methodology, while essentially the same bias correction/spatial disaggregation (BCSD) method as that used for NEX-GDDP, has been slightly updated to reflect the changes in experimental design between the two incarnations of CMIP.

**Table 1:** Variables included in the NEX-GDDP-CMIP6 archive.

Variable	Description	Units
hurs	Near-surface relative humidity	percentage
huss	Near-surface specific humidity	kg/kg
pr	Precipitation (including both liquid and solid phases)	kg/m^2^/s
rlds	Surface downwelling longwave radiation	W/m^2^
rsds	Surface downwelling shortwave radiation	W/m^2^
sfcWind	Surface wind speed	m/s
tas*	Near-surface air temperature	degrees K
tasmax*	Maximum near-surface air temperature	degrees K
tasmin*	Minimum near-surface air temperature	degrees K

*Downscaled tas (near-surface air temperature) is provided for models that did not provide tasmax and tasmin. Otherwise tas is derived from tasmax and tasmin.

The NEX-GDDP-CMIP6 archive will be useful for assessing trends in projected changes in climate at a variety of spatial and temporal scales, as well as for driving impacts and adaptation models that might require higher spatial and/or temporal resolution than might be available from other climate data products.

## Methods

The statistical downscaling algorithm used to create the NEX-GDDP datasets is a daily variant of the monthly (BCSD) method described in Wood *et al*.^2,3^. This variant method^4^ compares the original GCM output with corresponding climate observations over a common reference period and uses information derived from the comparison to adjust future climate projections so they are more consistent with the historical climate records and, presumably, more realistic for the spatial domain of interest. The algorithm also utilizes the spatial detail provided by observationally-derived datasets to interpolate the GCM outputs to higher-resolution grids.

The daily BCSD method described herein inherently assumes that the relative spatial patterns observed from the reference period will remain constant under future climate change. Other than the higher spatial resolution and bias correction, this dataset does not add information beyond what is contained in the original CMIP6 scenarios. While the frequency of periods of anomalously high and low values (i.e., extreme events) within each individual scenario are preserved, the downscaled values corresponding to extreme events might in some regions be underestimated due to diffusion over the spatial distribution resulting from the translation from low to high spatial resolution (e.g., Hwang and Graham^5^ Gutmann *et al*.^6^).

### Temperature  Pre-processing

Because the BCSD method does not explicitly adjust the trends (the slopes, in particular) in climate variables produced by GCMs, the monthly large-scale climate trends are extracted from the GCM temperature data. This is calculated as a 9-year running average for each individual month (e.g. the trend for all Januaries taken together). These trends are preserved and added back to the adjusted data after the bias-correction step.

### Bias correction

While recent studies^7,8^ have shown that the choice of observational data can impact the final downscaled output, to maintain consistency between dataset versions, the latest version of the same Global Meteorological Forcing Dataset (GMFD) for Land Surface Modeling^9^ that was used in creation of the original NEX-GDDP archive was used here. GMFD blends reanalysis data with observations and is available at spatial resolutions of 0.25 degrees, 0.5 degrees and 1.0 degrees, and at temporal resolutions of 3-hourly, daily, and monthly timesteps. Development of NEX-GDDP-CMIP6 utilized the 0.25-degree daily-averaged data for each variable of interest from 1960 to 2014.

**Fig. 3:**
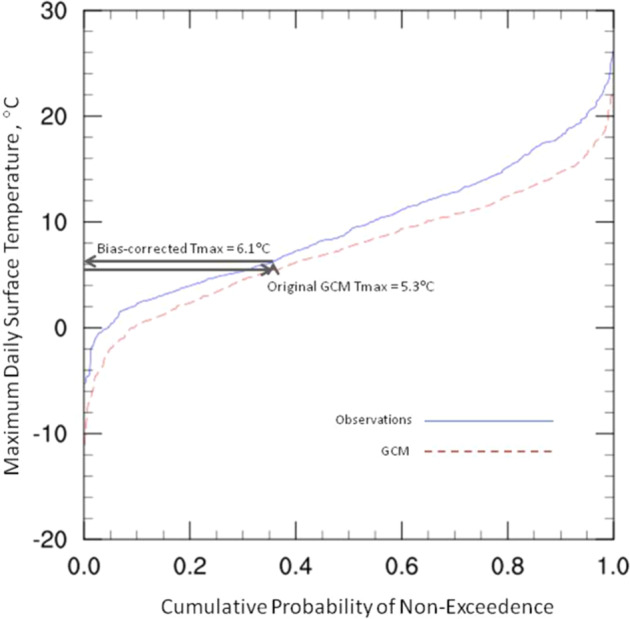
Example CDF for maximum daily near-surface temperature. Arrows illustrate the quantile mapping process used for bias correction. Adapted from Thrasher, *et al*., 2012.

These gridded observations were first interpolated to the resolution of the particular CMIP6 model being processed. A reference period of 1960–2014 was used as the basis for the cumulative distribution function (CDF), and each daily GCM value was evaluated using a +/− 15-day window over all years in the reference period to capture a full representation of candidate values for a particular variable. This resulted in 1,705 values (31 days over each of 55 years) to define each of the two CDFs for each value being corrected, one from the gridded observations and one from the GCM output from the historical experiment. The quantile associated with each original GCM value was calculated from that day’s GCM-based CDF distribution, then that quantile was used to calculate the corresponding value from the observation-based CDF distribution. This final value is the bias-corrected version of the GCM output. Figure 3 provides a visual example of this procedure. The one exception is the creation of bias-corrected minimum daily temperature, which is derived from the difference between the bias-corrected maximum daily temperature and the bias-corrected diurnal temperature range fields, as in Thrasher, *et al*.^4^.

### Spatial disaggregation

After all GCM values for a particular variable were bias-corrected, a spatial disaggregation was performed. This step essentially merges the observed historical spatial climatology with the relative changes at each time step simulated by the GCMs to produce the final results. A smoothed daily climatology over the reference period was created from the GMFD fields using a Fast Fourier Transform retaining three harmonics. This climatology was interpolated to the native grid of the GCM being processed and factored out of the bias-corrected output either through subtraction from the temperature variables or through division from the other variables. The residual fields were then bilinearly interpolated to the original 1/4-degree horizontal grid of the GMFD. At this point, the 1/4-degree climatology was factored back in either through addition to the temperature variables or through multiplication by the other variables. This results in the final downscaled product.

## Data Records

The NEX-GDDP-CMIP6 archive^10^ is made available through a CC-BY-SA 4.0 Creative Commons license and can be found at the NASA Center for Climate Simulation. Table 2 lists the current contents, including the variables available for each model, and notes on any inventory discrepancies between models. A single variant from each of the 35 models listed was used for downscaling. The historical experiment was downscaled over the years 1950–2014, while the SSP experiments were downscaled over the years 2015–2100. The downscaled output, on a standard 1/4-degree horizontal grid, is contained in yearly files in netCDF-4 classic format, with file sizes ranging from ~200–290MB. The current total data volume of the entire archive is approximately 30TB.

**Table 2:** CMIP6 models included in downscaled archive .

Key: Green = all experiments available; yellow = historical & some SSP(s) available; red = no data available.

*Original GCM output for hurs SSP245 missing year 2058

## Technical Validation

A quality control process was applied to all downscaled output to check that values fell within a realistic range. Table 3 shows the bounding values used to evaluate each variable. While values that fall outside these ranges are not necessarily erroneous, they should not occur very often. Indeed, these extraneous values occurred in less than 0.1% of values in the entire archive. Any additional validation required for a particular application of these data is left as an exercise for the end user.

**Table 3:** Bounds for validation of data values.

Variable	Lower bound	Upper bound
hurs	0%	102%
huss	0 kg/kg	0.04 kg/kg
pr	0 kg/m^2^/s	0.012 kg/m^2^/s
rlds	0 W/m^2^	500 W/m^2^
rsds	0 W/m^2^	500 W/m^2^
sfcWind	0 m/s	50 m/s
tas	200 K	340 K
tasmax	200 K	340 K
tasmin	200 K	340 K

## Usage Notes

The NASA Center for Climate Simulation (NCCS) provides open access to the data via a Thematic Real-time Environmental Distributed Data Services (THREDDS) Data Server (TDS). TDS is a web server that provides metadata and data access for scientific datasets, using OPeNDAP, OGC WMS and WCS, HTTP, and other remote data access protocols. Given the several methods of data access TDS provides, files can be download directly to a local system via the “HTTPServer” access method, or data can be subset and read directly off the server (see the NCCS TDS endpoint), which may be a more tenable option for users that cannot download the full files to their local systems due to the dataset size.

A simple option for data access is to download a set of files of interest from the TDS and then use some software package or application to read the netCDF files on the local system. Some tools can read subsets of the data off the server directly via the OpenDAP protocol (see the NCCS TDS endpoint). There are numerous high-level scripting languages, such as Python, and higher-level open source tools (e.g. GrADS, NCO, CDO, NCL, and Ncview) readily available to meet most user’s needs for data exploration and analytics.

The packaged NetCDF files are written with the CF-1.7 metadata conventions, which may provide an opportunity to read and work with the data in GIS oriented applications, such as the open source QGiS application. Users are encouraged to explore many of the tools and applications available.

## Data Availability

The NCL code used to generate the downscaled products can be found at https://github.com/bthrasher/daily_BCSD.
